# Cell therapy for full-thickness wounds: are fetal dermal cells a potential source?

**DOI:** 10.1007/s00441-015-2293-6

**Published:** 2015-10-09

**Authors:** J. J. Akershoek, M. Vlig, W. Talhout, B. K. H. L. Boekema, C. D. Richters, R. H. J. Beelen, K. M. Brouwer, E. Middelkoop, M. M. W. Ulrich

**Affiliations:** Department of Plastic, Reconstructive and Hand Surgery, Research Institute MOVE, VU University Medical Center, Amsterdam, The Netherlands; Association of Dutch Burn Centres, Zeestraat 27-29, 1941 AJ Beverwijk, The Netherlands; Department of Molecular Cell Biology and Immunology, VU University Medical Center, Amsterdam, The Netherlands; Euro Tissue Bank, Beverwijk, The Netherlands

**Keywords:** Fetal fibroblasts, Mesenchymal stem cells, Allogeneic/xenogeneic transplantation, Full-thickness wounds, Dermal substitutes

## Abstract

The application of autologous dermal fibroblasts has been shown to improve burn wound healing. However, a major hurdle is the availability of sufficient healthy skin as a cell source. We investigated fetal dermal cells as an alternative source for cell-based therapy for skin regeneration. Human (hFF), porcine fetal (pFF) or autologous dermal fibroblasts (AF) were seeded in a collagen–elastin substitute (Novomaix, NVM), which was applied in combination with an autologous split thickness skin graft (STSG) to evaluate the effects of these cells on wound healing in a porcine excisional wound model. Transplantation of wounds with NVM+hFF showed an increased influx of inflammatory cells (e.g., neutrophils, macrophages, CD4^+^ and CD8^+^ lymphocytes) compared to STSG, acellular NVM (Acell-NVM) and NVM+AF at post-surgery days 7 and/or 14. Wounds treated with NVM+pFF presented only an increase in CD8^+^ lymphocyte influx. Furthermore, reduced alpha-smooth muscle actin (αSMA) expression in wound areas and reduced contraction of the wounds was observed with NVM+AF compared to Acell-NVM. Xenogeneic transplantation of NVM+hFF increased αSMA expression in wounds compared to NVM+AF. An improved scar quality was observed for wounds treated with NVM+AF compared to Acell-NVM, NVM+hFF and NVM+pFF at day 56. In conclusion, application of autologous fibroblasts improved the overall outcome of wound healing in comparison to fetal dermal cells and Acell-NVM, whereas application of fetal dermal fibroblasts in NVM did not improve wound healing of full-thickness wounds in a porcine model. Although human fetal dermal cells demonstrated an increased immune response, this did not seem to affect scar quality.

## Introduction

Deep partial and full-thickness burn wounds often result in hypertrophic scars. Although burn wound care has improved over the years, scarless wound healing is still beyond reach. One of the causes that lead to hypertrophic scarring of burn wounds is partial or complete loss of the dermis. The use of collagen-based dermal substitutes to replace the lost and/or damaged dermis has been shown to improve the outcome of wound healing. Examples of this have been described by Bloemen et al. (2010) and Ryssel et al. (2008), who showed that transplantation of acellular collagen-based dermal substitutes with split thickness skin grafts (STSG) on top of full-thickness burn wounds improved scar quality. A smoother skin (less relief) and better pliability were observed, even 12 years post-surgery, compared to the control which received only STSG (Bloemen et al. 2010). Despite improved scar quality when using acellular dermal substitutes, the optimal result of full skin regeneration is still not achieved. Cell-based therapy is one of the strategies that may further improve skin regeneration. Indications for this have been found in the use of autologous dermal cells which were shown to improve the wound healing and/or scar formation of burn wounds. Lamme et al. (2000) demonstrated in a porcine full-thickness wound model that addition of autologous dermal cells to a dermal substitute can reduce scarring in vivo, as improved scar quality in terms of reduced redness, contraction and relief was observed compared to the acellular dermal substitute. Furthermore, dermal substitutes seeded with autologous dermal cells also significantly reduced alpha smooth muscle actin (αSMA) expression in the wound area compared to acellular dermal substitutes. Expression of αSMA is indicative of the presence of myofibroblasts and contraction in scar tissue of burn wounds. Improved healing of burn wounds after application of substitutes containing autologous dermal cells was also shown clinically in a case report by Wisser and Steffes (2003).

In contrast to allogeneic cells, the advantage of using autologous cells is that they will not elicit a host versus graft (rejection) reaction upon transplantation. On the other hand, one of the major hurdles with autologous cell-based therapies is to obtain sufficient cells in a limited amount of time. Especially, patients with extensive burn wounds have limited availability of healthy skin for harvesting autologous dermal cells. Moreover, expansion of the autologous cells to a sufficient number may take up to 3 weeks (Hata 2007; Tan et al. 2014) and is associated with high costs. To overcome these hurdles, an alternative cell source is sought for use in cell-based therapies. One of the cell types that has been proposed are mesenchymal stem cells (MSCs). MSCs are known for their multipotency (Dominici et al. 2006; Maxson et al. 2012). It has been reported that direct transplantation of MSCs, or seeded in dermal substitutes, can have beneficial effects on wound healing in vitro and in vivo (Laverdet et al. 2014; Maxson et al. 2012). An in vivo animal study by Liu et al. (2008) showed reduced wound contraction, improved wound healing and better vascularization and re-epithelialization after transplantation of collagen–glycosaminoglycan substitutes seeded with autologous bone marrow-derived MSCs in partial thickness burn wounds using a porcine model. Several tissues can serve as a source for MSCs, such as bone marrow, fat, gingiva, and dermis, as well as fetal tissue (umbilical cord, placenta) (Laverdet et al. 2014). However, the accessibility, cell number and availability can vary between the different tissues.

Fetal dermal cells which have MSC characteristics (Chinnici et al. 2014; Tan et al. 2014; Young et al. 2001) have been indicated as a promising cell source for cell-based therapy for skin regeneration. An essential advantage is that fetal skin cells have a better regenerative and higher proliferative capacity compared to adult cells (Ferguson et al. 1996; Kishi et al. 2012; Rolfe and Grobbelaar 2012; Yates et al. 2012). Fetal wounds are known to heal faster, and with nearly no scarring up to the middle of the third trimester (Ferguson et al. 1996; Satish and Kathju 2010). They also seem to have a higher recovery rate after cryopreservation compared to adult cells (Applegate et al. 2009; De Buys Roessingh et al. 2006). In addition, fetal cells have a low immunogenicity which is an advantage with respect to allograft transplantation and rejection. MHC molecules play an important role in this, since MSCs (Ryan et al. 2005), but also fetal dermal cells (Chinnici et al. 2014) (<14 weeks of gestation for humans) have a very limited expression of MHC class II molecules on their cell surface. The expression of MHC class II molecules remains absent after culturing of fetal dermal cells until passages 21–28 (Chinnici et al. 2014; Hohlfeld et al. 2005). Furthermore, MSCs have been shown to modulate CD4^+^ and CD8^+^ T lymphocyte responses in in vitro co-cultures (Barry et al. 2005; Ryan et al. 2005). In vitro studies showed, for example, that MSCs inhibited T lymphocyte proliferation, increased CD4^+^ CD25^+^ T lymphocyte (T_regs_) populations, suppressed CD4^+^ and CD8^+^ lymphocyte activity and created an immunosuppressive environment (Barry et al. 2005; Le Blanc 2003; Ryan et al. 2005).

All these advantages might allow the creation of a well-characterized fetal skin cell bank which could be used for cell-based therapies. Development of such an “off-the-shelf” product would remedy the cell number deficiency and decrease culture time for cell-based dermal substitutes. Applegate and colleagues have already investigated the establishment of a fetal skin cell bank (Applegate et al. 2009; De Buys Roessingh et al. 2006). They were able to create several million constructs out of just one 4 cm^2^ fetal dermal tissue biopsy (Applegate et al. 2009; Hohlfeld et al. 2005). Moreover, they used constructs with fetal skin cells for burn wounds in children and observed faster wound healing and less scarring (Hohlfeld et al. 2005). However, fetal constructs were topically administered and refreshed every dressing change. There are two explanations for the results of this study: migration of fetal dermal cells into the wound bed, or that these cells only provided growth factors, chemokines and cytokines by paracrine signaling (Chen et al. 2008; Gnecchi et al. 2008; Hocking and Gibran 2010). Furthermore, the burn wounds of the children treated with the fetal dermal constructs seemed relatively small, and were possibly not completely full-thickness wounds (Scott and Tredget 2005). Therefore, the beneficial effects of dermal substitutes containing fetal dermal cells for the application in full-thickness (burn) wounds remain to be elucidated.

The aim of our study was to investigate the effects of fetal dermal cells on wound healing and scarring of full-thickness wounds. A porcine full-thickness wound model was used to study the effect of a porcine collagen-based dermal substitute (Novomaix, NVM) seeded with human (xenogeneic) or porcine (allogeneic) fetal dermal cells in combination with a STSG on wound healing and scar formation. These treatments were compared to NVM seeded with autologous dermal fibroblasts. We investigated the wound healing and scar characteristics, as well as the inflammatory response of these different treatment modalities.

## Materials and methods

### Fetal and autologous dermal fibroblast isolation

#### Human fetal fibroblasts

Human fetal fibroblasts were isolated from fetal skin (derived from extremities) after pregnancy termination; the age of the fetus was 12 weeks (one donor). Fetal skin was obtained with the informed and written consent of the pregnant woman.

Skin tissue was washed with phosphate-buffered saline (PBS) and cut into small pieces for enzymatic treatment. Tissue was digested using 0.25 % (w/v) collagenase A and 0.25 % (w/v) dispase II (Roche Diagnostics, France) in PBS for 1–2 h at 37 °C under agitation (van den Bogaerdt et al. 2002). Digested tissue was washed with PBS containing 1 % fetal calf serum (FCS) (HyClone FetalClone III; Thermo Scientific, Etten-Leur, The Netherlands) and filtered over an open filter chamber (Beldico, Duiven, The Netherlands) to remove large tissue debris particles. Cells were pelleted by centrifugation for 10 min at 385*g*. Cell pellets were resuspended in fibroblast culture medium (FBM) which consisted of DMEM (Gibco, Paisley, UK) supplemented with 10 % v/v FCS, 1 mM L-glutamine, and the antibiotics penicillin (100 IU/mL) and streptomycin (100 mg/mL) (Gibco). The cell suspension was filtered through a 70-μm cell strainer (Falcon; Becton Dickinson, Mountain View, CA, USA) to remove any remaining tissue debris. Cells were pelleted (267*g*, 10 min), resuspended in FBM and seeded with a cell density of 3 × 10^6^ per 175-cm^2^ tissue culture flask (1.7 × 10^4^ per cm^2^). From human adult dermis, an average of 1 × 10^6^ cells/g tissue can be obtained and for human (and porcine) fetal skin an average of 20 × 10^6^ cells/g tissue. Cell doubling times for human adult fibroblasts is 4.6 days and for fetal fibroblasts 2.3 days. Cells were cultured until a sufficient amount of cells was obtained. Cells of passages P1–P4 were used in the experiment.

#### Porcine fetal and autologous fibroblasts

For the isolation of porcine fetal dermal cells, unborn piglets were isolated from a 60-day pregnant Yorkshire pig. Skin was taken from flanks of the piglets. For the isolation of autologous (adult) fibroblasts, two sheets (5 × 20 cm, 0.3 mm) of split thickness autologous skin were taken from the back of the animals (from animals in which later full-thickness wounds were created) with an electrical dermatome. This was performed 1 week before transplantation. Isolation of the porcine autologous and fetal fibroblasts was performed as described above in ‘human fetal fibroblasts’. Porcine fetal and autologous fibroblasts were cultured and seeded into Novomaix (NVM) dermal substitutes.

### Cell characterization

hFF, pFF and AF were seeded onto glass slides with a density of 50,000 cells/slide. After 2–3 days of culture, cells were fixed with 4 % formaldehyde for 15 min at RT. Cells of passages P2–P5 were used for immunohistochemistry. Cells were pretreated with 0.5 % Triton X-100. Next, cells were stained for heat shock protein 47 (HSP47), which is used as a fibroblast marker, and αSMA for 1 h at RT (see Table 4 for antibodies and dilutions). Subsequently, slides were incubated for 45 min at RT in the dark using goat-anti-mouse Alexa488 (1:500, A21424; Life Technologies, VWR, Amsterdam, The Netherlands) and goat-anti-mouse Alexa555 (1:500, A11017; Life Technologies) respectively as secondary antibodies.

### Dermal substitute Novomaix

Novomaix (NVM) (Matricel, Herzogenrath, Germany) collagen–elastin scaffolds (average pore size 100 μm) were used as dermal substitutes. The highly purified collagen and elastin fibers of this scaffold are of porcine origin. Characteristics and features of NVM have been described previously by Boekema et al. (2014). Three days before surgery, NVM was cut into pieces of 3.5 × 3.5 cm and prewetted with FBM. Human (hFF) or porcine (pFF) fetal or autologous (AF) fibroblasts were seeded on top of the dermal substitutes with a cell density of 5 × 10^5^ fibroblasts/cm^2^. The cell suspension was absorbed by the dermal substitute. Two hours of incubation at 37 °C was implemented to allow fibroblasts to attach to NVM, and thereafter 20 mL of FBM culture medium containing 65 μg/mL vitamin C (Sigma Aldrich, Steinheim, Germany) was added per dermal substitute. Culture medium was replaced every day with medium with freshly added vitamin C until the day of surgery. The acellular NVM (Acell-NVM) was treated similar to the cell-seeded NVM, only all procedural steps were without cells. Acellular and cell-seeded substitutes were transplanted on excised full-thickness wounds on the day of surgery. Treatments were randomly appointed to the wounds per animal as described in Table 1. Two wounds per animal received the same treatment. The treatment with STSG alone is comparable to the treatment that is standard in the clinic. Furthermore, addition of AF to the dermal substitute NVM was also used as a control because these cells have already been shown to improve scar quality.

**Table 1:** Treatment modalities of cell-seeded and control treatments

Treatment	Abbreviation treatment	Total number of wounds
Split thickness skin graft	STSG	12
Novomaix (NVM) (acellular) + STSG	Acell-NVM	12
Human fetal dermal fibroblasts + NVM + STSG	NVM+hFF	6
Porcine fetal dermal fibroblasts + NVM + STSG	NVM+pFF	6
Autologous fibroblasts + NVM + STSG	NVM+AF	12

### Animal experiments, operative and bandaging procedures

The institutional Animal Experiments Committee of the VU University Medical Center Amsterdam, The Netherlands, approved the experimental protocols according to governmental and international guidelines on animal experimentation. The pigs received a week’s acclimatization period before the start of the experiment. The pigs were individually housed, fed twice a day and had access to water ad libitum. In total, six female Yorkshire pigs were used. Animals had an average body weight of 31 kg at the start of the experiment and an average of 67 kg at the end.

#### Animal sedation/anesthesia/analgesia

Twenty minutes before the start of the surgical procedure, the animals were sedated with a combination of Ketamine 10 mg/kg, Dormicum (Midazolam) 0.5 mg/kg and Atropine 0.5–1.0 mg by intramuscular (i.m.) injection. Anesthesia during the tattoo, biopsy, and bandage-change procedures was induced and maintained by 35 % O_2_, 65 % NO_2_ and 1.5–2.5 % Isoflurane via a mouth cap. Analgesic Novum 20 (Meloxicam, 0.04 mg/kg) was administered i.m.

Before the surgery (creation of full-thickness wounds, application of dermal substitutes and STSG transplantations), full anesthesia was induced using Etomidate lipidor via intravenous (i.v.) injection (sedation), Dormicum (Midazolam) 15 mg, and as analgesia Fentanyl 200 μg and muscle relaxant Pavulon (Pancuronium bromide) 6 mg (i.v.). During surgery, the anesthesia was maintained by i.v. injection of Dormicum 0.5 mg/kg/h, Fentanyl 6.5 μg/kg/h and Pavulon 0.27 mg/kg/h. Furthermore, artificial respiration with 45–50 % O_2_ and 1.5–2.5 % Isoflurane was provided when the animals received full anesthesia. Vital functions such as CO_2_ expiration concentration, blood gas values, heart rate and temperature were monitored during surgery. Direct post-operative analgesia was administered by i.m. injection of Buprenorphine 0.3 mg. After the surgical procedure, 5 μg/kg Buprenorphine was administered at least once, or every 10–12 h when there was indication of pain. Metacam was orally administered once a day for a period of 2 weeks.

#### Surgical procedure

Surgical procedures of these animal experiments were performed using the porcine excisional wound model described by Middelkoop et al. (2004). In short, 1 week before the creation of the full-thickness wounds, a grid of 7 × 7 cm squares was tattooed on both flanks of the pigs. This grid is used to correct for the growth of the animal. At the day of surgery, seven full-thickness, 2.7-mm-deep wounds of 3 × 3 cm squares were created on each flank of the pig. Autologous STSGs (0.3 mm thickness) were obtained from the wound sites using an electrical dermatome (Humeca, Enschede, The Netherlands). STSG were meshed (Humeca) in a 1:3 ratio and kept moisturized in sterile PBS-soaked gauzes until use. Wounds were excised with a scalpel to full-thickness (until the subcutaneous fat layer). Twelve of 14 full-thickness wounds per animal were transplanted with a dermal substitute (NVM), with or without cells, and covered with a meshed STSG. Two of the 14 wounds per animal received only an STSG. Dermal substitutes and STSGs were fixed with staples (3M, Delft, The Netherlands). The different treatments were randomized over the seven wounds on each flank. Two wounds per animal received the same treatment. Three of the seven wounds per flank received other treatments, which will not be described in this article. Therefore, the number of wounds described in this article is in total 48. Wounds were dressed with adhesive Alleveyn (gentle border 10 × 10 cm; Smith & Nephew, Hull, UK) to protect against mechanical trauma and contamination. Sterile gauzes (45 × 70 cm) were used to cover the Alleveyn bandages, and were fixed with adhesive bandage Curafix (Lohmann & Rauscher, Neuwied, Germany) and the elastic stocking Tubigrip was used (Klinion; Mediq Medeco, Oud-Beijerland, The Netherlands).

### Macroscopic wound evaluation and dressing changes

Wounds were macroscopically evaluated during dressing changes at 4, 7, 14, 21 days, and at the end of the experiment at 56 days post-surgery. Macroscopic evaluation of the wounds was performed by 2–3 independent trained observers who were blinded for the treatment. Parameters used in the macroscopic evaluation were obtained and adjusted from the observer part of the Patient and Observer Scar Assessment Scale (POSAS). The POSAS is a validated method used for scar evaluation in patients in the clinic (Draaijers et al. 2004; van der Wal et al. 2012). Wounds were evaluated for graft take, wound color, wound closure, wound thickness, skin smoothness and wound pliability (Table 2). At the end of the experiment (day 56), each wound was given an overall observer score for scar quality (Table 2). This scar quality score was based on all scored parameters (such as contraction, redness, thickness and relief) at day 56.

**Table 2:** Parameters for macroscopic wound evaluation

Parameter	Score	Description
Graft take	Percentage (%) of vital graft from total graft	I.e., viability of the graft (pink color) and adherence to the wound bed (%)
Wound closure	Percentage (%) of total wound area that is covered by epithelium	Re-epithelialization of the wound area
Wound color	Scale 0 to 5	Compared to unaffected skin (pink, =0), + 5 = purple/red
Wound thickness (depth)	Scale −5 to 5	Level compared to normal epidermis level (=0), −5 = deep wound, +5 = raised wound
Distortion (compared to original wound shape)	Scale 0 to 5	0 = original size wound square, 5 = severe star-shaped wound area
Observer score (scar quality)	Scale 1 to 10	Compared to unaffected skin (1 = unaffected skin)

Digital pictures of the wounds were taken on days 0, 4, 7, 14, 21 and 56. To measure contraction of the wound at days 0, 21 and 56, the wound edges and tattoo grid were traced onto a transparent sheet. Contraction was determined by planimetry (NIS Elements, v.3.1; Nikon, Badhoevedorp, The Netherlands) and corrected for the growth of the animal by calculating the wound area as a ratio of the area of the tattoo grid. This was subtracted from the original wound/tattoo grid area relation of day 0. Contraction is expressed as percentage contraction of the wound compared to day 0 which was set at 0 % (no contraction). The measurement of wound contraction is explained in Lammers et al. (2011: fig. 2). Two punch biopsies (Ø 4 mm) of the wounds were taken on days 4, 7, 14 and 21, in a systematic order. At the end of the experiment (day 56), the animals were euthanized with 30 mL pentobarbital sodium i.v., and subsequently a large biopsy across the center of the wound was taken. Wound biopsies were fixed in Kryofix (48 % ethanol, 7 % polyethylene glycol (PEG)-300) and stored at 4 °C, or snap-frozen in liquid nitrogen and stored at −80 °C until further analysis.

### Microscopic wound evaluation

Kryofixed tissue biopsies were dehydrated, embedded in paraffin and cut into sections of 5-μm thickness. For (immuno)histochemical stainings, sections were deparaffinized and rehydrated. A hematoxylin (Mayers; DAKO, Glostrup, Denmark) and eosin (Sigma-Aldrich) staining was performed to evaluate dermal substitute presence, epidermis, number of foreign body giant cells (FBGC) and granulation tissue thickness (Table 3). Granulation thickness was measured using digital image analysis (NIS Elements, v.3.1).

**Table 3:** Parameters for microscopic wound evaluation

Parameters	Score	
Presence of dermal substitute	Scale 0 to 3	0 = no dermal substitute remnants, 3 = intact dermal substitute structure
Epidermis	Scale 0 to 3	0 = no epidermis, 3 = hyperproliferation of keratinocytes
Foreign body giant cells (FBGC)	Number of FBGC cells in wound area of the tissue section	
Thickness granulation tissue	Micrometer (μm)	Distance from start of dermis to subcutaneous fat layer

Presence of myofibroblasts in wound tissue sections was assessed by αSMA expression using an antibody against αSMA (Table 4). Sections were stained with an antibody directed against CD31 to study the presence of blood vessels (Table 4). Neutrophils were stained using an antibody directed against MPO (Table 4). To investigate the treatment effects on the immunological response, frozen sections (5 μm) were stained for immune cells: macrophages (CD163), CD4^+^ and CD8^+^ lymphocytes (Table 4).

**Table 4:** Primary antibodies

Primary antibody	Clone	Dilution	producer
Anti - αSMA	1A4	1:500	DAKO, Glostrup, Denmark
Anti - CD31	SP38 (M3380)	1:150	Abbiotec, San Diego, CA, USA
Anti - MPO	A0398	1:1200	DAKO, Glostrup, Denmark
Anti - CD163	MAC2.48	1:1000	IQproducts, Groningen, The Netherlands
Anti - CD4	MIL17 (MCA1749)	1:50	AbD Serotec, Puchheim, Germany
Anti - CD8	MIL12 (MCA1223)	1:100	AbD Serotec, Puchheim, Germany
Anti - HSP47	M16.01A1	1:500	Enzo Life Sciences, Raamsdonksveer, The Netherlands

Slides were incubated with secondary antibodies BrightVision Poly-HRP anti-mouse or anti-rabbit (Immunologic, Duiven, The Netherlands). The substrate 3,3′-Diaminobenzidine (DAB) (Immunologic) was used to visualize the staining. Tissue sections were counterstained with hematoxylin. We determined the wound area positive for αSMA expression by selection of the DAB signal in the tissue using NIS Elements (Nikon). The area of the DAB signal was expressed as a fraction of the total wound area. Immune cells were scored for presence on a scale of 0 (not present) to 5 (severe influx). All microscopic analyses were performed blinded to the treatment and scored by at least two trained observers.

### Statistics

All statistical analyses were performed with SPSS (v.21.0 MS Windows; SPSS, Chicago, IL, USA). Statistically significant differences between treatment groups were determined by using the nonparametric Kruskal–Wallis test. A two-tailed Mann–Whitney *U* test was used as a post hoc test. A *p* value of <0.05 was considered statistically significant. Graph bars represent the mean and the standard deviation per treatment.

## Results

### Mesenchymal stem cell characteristics of fetal dermal cells

**Fig. 1:**
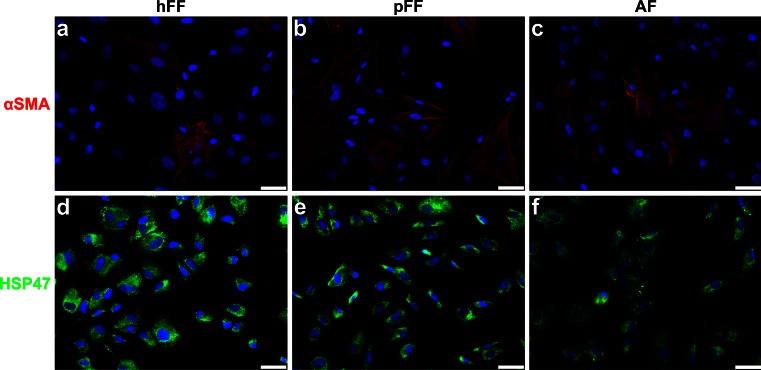
Cell characterization by αSMA and HSP47 expression in hFF, pFF and AF (porcine), cells cultured on glass for 2–3 days and stained for DAPI (*blue*), HSP47 (*green*) and αSMA (*red*). **a**–**c** All cell sources showed a number of αSMA positive cells in the cell population. All αSMA positive cells showed stress fiber formation. **d**–**f** HSP47 stained the cytoplasm of all cells, indicating their fibroblastic phenotype. *Scale bars* 50 μm

The presence of mesenchymal stem cells in a fetal dermal cell population and their characteristics were assessed by flow cytometric analysis and differentiation capacity. Human fetal dermal cells were positive for CD105, CD73 and CD90, and were negative for CD14, CD31, CD34, CD45, CD79a and HLA-DR (data not shown). Both human and porcine fetal dermal cells were able to differentiate into osteoblasts, chondrocytes and adipocytes (data not shown). These results meet the criteria as defined by Dominici et al. (2006)*.* Phenotypical characterization of adult MSCs derived from different sources has been described by van den Bogaerdt et al. (2009). AF, hFF and pFF cultured on glass slides showed similar staining of the cytoplasm for HSP47 (Fig. 1d-f). αSMA expression in these cells was also similar for AF and pFF; however, the αSMA expression in hFF seemed slightly lower (Fig. 1a-c). αSMA expression by these cells was represented as a stress fiber phenotype.

### Autologous fibroblasts in collagen-based dermal substitutes improved scar quality

**Fig. 2:**
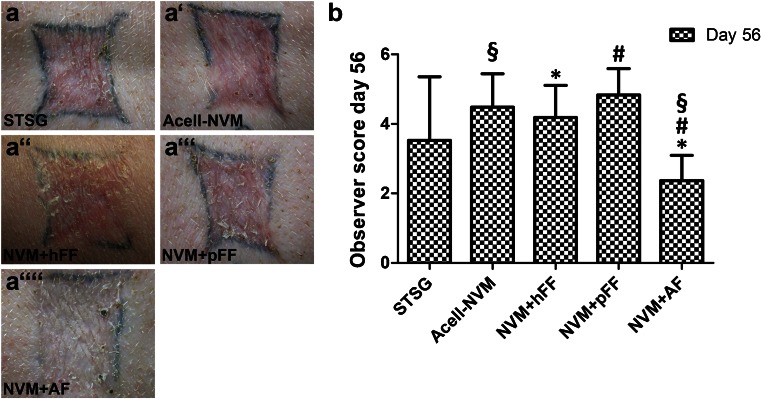
Macroscopic evaluation of treated wounds at day 56. **a** Representative photographs of wounds per treatment. **b** Scar quality (overall observer score) as determined by scoring parameters of the POSAS scale. Scar quality was improved (represented by lower scores) in wounds transplanted with NVM+AF (*n* = 12 wounds) compared to Acell-NVM (§, *n* = 12 wounds), NVM+hFF (*, n = 6 wounds) and NVM+pFF (#, *n* = 6 wounds). Statistical significance is indicated by *symbols* (Mann–Whitney *U* test, *p* <0.05). *STSG* Split thickness skin graft, *Acell-NVM* Novomaix+STSG, *NVM+hFF* Novomaix containing human fetal fibroblasts + STSG, *NVM+pFF* Novomaix containing porcine fetal fibroblasts + STSG, *NVM+AF* Novomaix containing autologous fibroblasts +STSG

Scars of the differently treated wounds were macroscopically evaluated 56 days post-surgery using an adapted POSAS scar assessment scale (Table 2). A lower score represents a better scar quality, indicating closer similarity to unaffected skin. Figure 2a illustrates a macroscopic view of the scars at 56 days post-surgery. Wounds transplanted with NVM+hFF, NVM+pFF, Acell-NVM or STSG demonstrated a more star-shaped scar (distortion) compared to wounds treated with NVM+AF. The overall observer scores (scar quality) of the NVM+hFF and NVM+pFF group were similar to STSG and Acell-NVM treatment (Fig. 2b). Wounds treated with NVM+AF showed an improved scar quality compared to Acell-NVM, NVM+hFF and NVM+pFF at post-surgery day 56 (Fig. 2b). No differences of other macroscopic (Table 2) and microscopic (Table 3) evaluation scores were observed between the different treatments.

### Collagen-based dermal substitutes seeded with autologous fibroblasts reduced scar contraction and αSMA expression

**Fig. 3:**
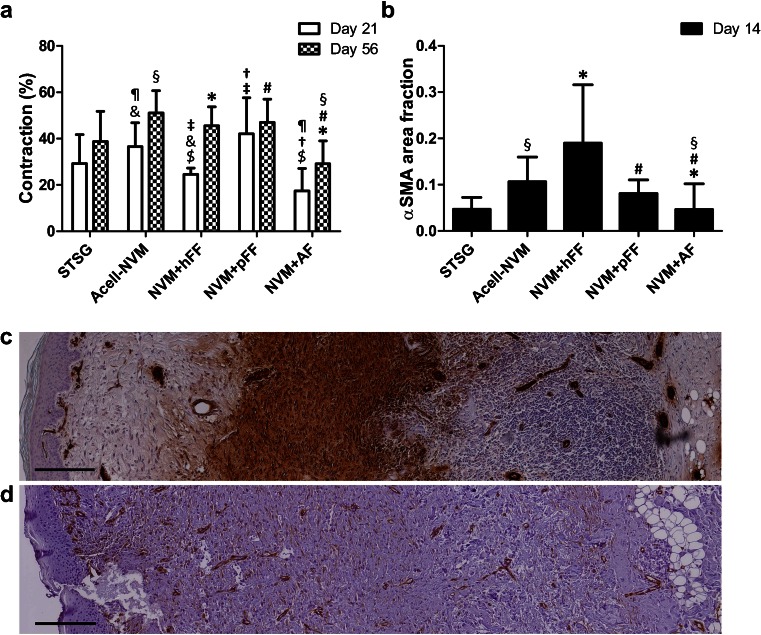
Wound contraction and αSMA^+^ myofibroblasts in the wound area. **a** Planimetric analysis of wound area to determine wound contraction at day 21 and day 56. Contraction is expressed as percentage contraction of total wound area. NVM+AF (*n* = 12) showed a reduction in the contraction of the wound compared to Acell-NVM (¶, §, *n* = 12), NVM+hFF ($, *, *n* = 6) and NVM+pFF (†, #, *n* = 6). Statistical significance is indicated by *symbols* (Mann–Whitney *U* test, *p* < 0.05). **b** An immunohistochemical staining for αSMA was performed to detect the presence of myofibroblasts in the wound at post-surgery day 14. αSMA expression of myofibroblasts was calculated as fraction of the wound area. Wounds transplanted with hFF showed an increased expression of αSMA in the wound area compared to NVM+AF (*) at day 14. NVM+AF showed a reduced αSMA expression in the wound in comparison with Acell-NVM (§), NVM+hFF (*) and NVM+pFF (#). **c**, **d** Representative pictures of wound tissue sections stained for αSMA (DAB, in *brown*) at day 14. Full-thickness wound transplanted with NVM+hFF (**c**) showed higher αSMA expression in the wound area compared to NVM+AF (**d**)

Excessive wound contraction is a frequently encountered characteristic of burn wound scars. Contraction of the wound was macroscopically evaluated (see description above), but also determined by planimetric analysis at days 21 and 56 post-surgery (Fig. 3a). Increased contraction was noted for wounds treated with NVM+hFF versus Acell-NVM at day 21. Full-thickness wounds transplanted with NVM+hFF or NVM+pFF showed statistically significantly more contraction compared to NVM+AF at post-wounding days 21 and 56 (Fig. 3b). A lower wound contraction was only observed in wounds transplanted with NVM+hFF compared to NVM+pFF at day 21. Furthermore, addition of AFs to a dermal substitute reduced contraction of full-thickness wounds in comparison Acell-NVM (day 21 and 56). Overall, contraction increased over time.

The presence of myofibroblasts in the wound area was analyzed by immunohistochemical staining of αSMA at day 14. Figure 3b illustrates that a statistically significantly larger fraction of the total wound area was αSMA positive in wounds transplanted with NVM+hFF or pFF compared to NVM+AF. Wounds of NVM+hFF showed a clear αSMA-positive expression in the granulation tissue (Fig. 3c), while αSMA-positive expression was significantly less in wounds of NVM+AF (Fig. 3d) at day 14. Induction of αSMA expression in the wound area tended to be less in wounds treated with NVM+pFF compared to NVM+hFF (*p* = 0.055). It is noteworthy that the addition of AF to NVM significantly reduced the αSMA expression in the wound area compared to Acell-NVM at day 14.

### Increased influx of inflammatory cells after transplantation of fetal fibroblasts

NVM seeded with hFFs or pFFs did not improve the macroscopic scar characteristics or scar quality compared to the Acell-NVM treatment. Since inflammation can influence scar formation, the presence of inflammatory cells such as neutrophils, macrophages, CD4^+^ and CD8^+^ T lymphocytes in the wound area was investigated.

**Fig. 4:**
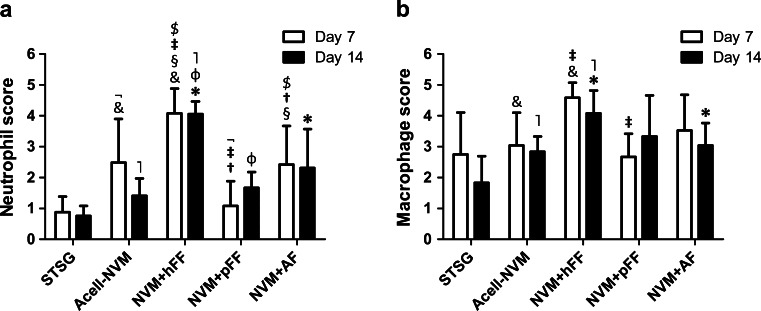
Neutrophil and macrophage influx in full-thickness wounds. Immune cells were detected with immunohistochemistry and scored blinded upon grades of influx from 0 (none) to 5 (massive influx). **a** Neutrophils were stained with an antibody directed against myeloperoxidase (MPO). Increased neutrophil scores were observed in wounds transplanted with NVM+hFF compared to Acell-NVM (&, ˥), NVM+pFF (‡, Ø) and NVM+AF ($, *) (days 7 and 14). **b** Influx of macrophages (antibody MAC 2.48) in wounds was scored per treatment for post-surgery day 7 and 14. NVM+hFF induced the macrophage influx in the wound area compared to Acell-NVM (&, ˥, days 7 and 14) and NVM+AF (*, day 14). Statistical significance indicated by *symbols* (Mann–Whitney *U* test, *p* < 0.05)

Neutrophils and macrophages play an important role in clearing of infections and tissue debris (e.g., dead cells). An increased influx of neutrophils was observed upon transplantation of NVM+hFF compared to all the other treatments at days 7 and 14 (Fig. 4a). The influx of neutrophils remained at the same level until day 14 in the NVM+hFF group. Wounds treated with NVM+pFF showed a much lower influx of cells than NVM+hFF (days 7 and 14). In addition, NVM+pFF showed a lower neutrophil influx compared to Acell-NVM and NVM+AF at day 7. Furthermore, statistically significant lower neutrophil scores were observed in wounds treated with NVM+AF compared to NVM+hFF at both post-surgery days 7 and 14 (Fig. 4a).

Macrophage scores in Fig. 4b illustrate that full-thickness wounds transplanted with NVM+hFF showed statistically significant higher macrophage influx compared to Acell-NVM (days 7 and 14), NVM+pFF (day 7) and NVM+AF (day 14). No differences in macrophage influx were detected when NVM+pFF and NVM+AF were compared with Acell-NVM at day 14. Overall, a declining trend in macrophage score was observed for all treatments at day 14 versus day 7, except for the NVM+pFF group.

**Fig. 5:**
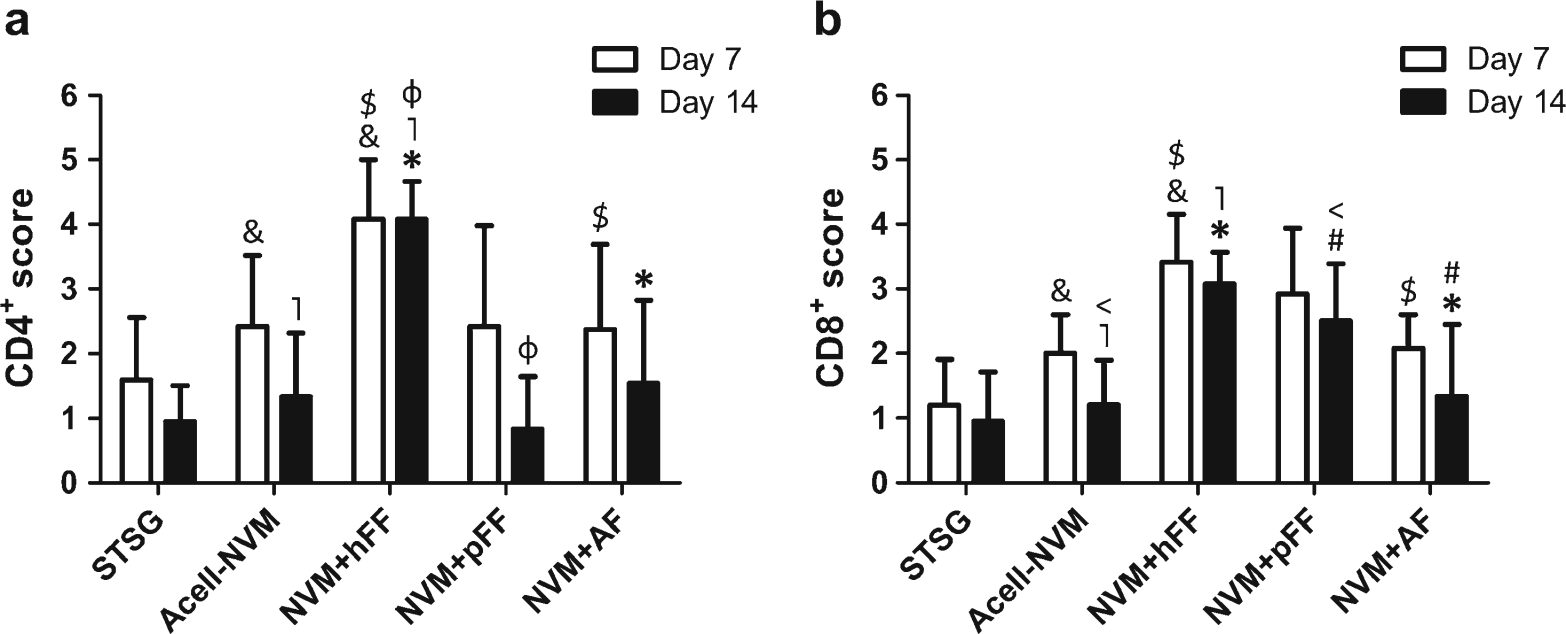
Influx of CD4^+^ and CD8^+^ lymphocytes in full-thickness wounds. The influx of CD4^+^ and CD8^+^ lymphocytes in the wounds was scored blinded for each treatment group (on a scale of 0 (none) to 5 (massive influx)). **a** Score of CD4^+^ lymphocyte influx in the wound area. A statistically significant higher influx of CD4^+^ lymphocyte was observed in wounds transplanted with NVM+hFF compared to Acell-NVM (&, ˥), and NVM+AF ($, *) (days 7 and 14). **b** Influx scores of CD8^+^ lymphocytes infiltrated into the wound area. Higher influx of CD8^+^ lymphocytes was observed in wounds treated with NVM+hFF or NVM+pFF in comparison to Acell-NVM (vs. hFF: &, ˥; vs. pFF: <) and NVM+AF (vs. hFF:$, *; vs. pFF: #). Statistical significance is indicated with *symbols* (Mann–Whitney *U* test, *p* < 0.05)

One of the most important issues with allogeneic and xenogeneic transplantation is the rejection of graft cells by the receiver. Both CD4^+^ and CD8^+^ lymphocytes play a role in this process. Especially, cytotoxic CD8^+^ lymphocytes are able to recognize foreign MHC molecules. Full-thickness wounds transplanted with NVM+hFF induced the influx of CD4^+^ and CD8^+^ lymphocytes in comparison to Acell-NVM and NVM+AF at days 7 and 14 post-surgery (Fig. 5). Enhanced influx of CD4^+^ lymphocytes was observed for NVM+hFF compared to NVM+pFF at day 14 (Fig. 5a, b). Remarkably, CD4^+^ lymphocyte scores were reduced for all treatment groups at day 14, except for the hFF group. The CD4^+^ lymphocyte score for NVM+hFF treatment remained at the same level as day 7. This suggests that the CD4^+^ lymphocyte response is prolonged when wounds are transplanted with NVM containing hFF. Besides an induction in the influx of CD4^+^ lymphocytes within the NVM+hFF group, an elevation in the CD8^+^ lymphocyte influx was also noticed. Both NVM+hFF and NVM+pFF groups induced the CD8^+^ lymphocyte influx compared to Acell-NVM at days 7 and 14 (Fig. 5). Fourteen days post-surgery, the scores of CD8^+^ lymphocytes of NVM+hFF and NVM+pFF remained at a similar level as at day 7. At day 14, no differences in CD8^+^ lymphocyte scores were observed in wounds treated with NVM+AF in comparison with Acell-NVM. Interestingly, NVM+hFF (days 7 and 14) and NVM+pFF (day 14) transplanted wounds showed a higher CD8^+^ lymphocyte increase than NVM+AF transplanted wounds.

## Discussion

Fetal dermal cells have been proposed as a potential alternative cell source replacing autologous cells in cell-based transplantation therapies. This is largely based on their multipotency, hypothesized hypo-immunogenicity and high expansion rate. It is important to gain knowledge about these fetal dermal cells and their behavior in a wound environment, before they can be used as cell-based therapy in the clinic.

Most importantly, this study showed that transplantation of excised full thickness wounds with either NVM+hFF or NVM+pFF did not improve nor deteriorate scar quality in comparison to an acellular NVM substitute in the porcine model after 56 days. Wounds that were transplanted with NVM containing autologous fibroblasts (AF) demonstrated a better scar quality 56 days post-surgery than wounds treated with NVM without cells or with fetal cells. Full-thickness wounds treated with NVM+AF also resulted in a reduced wound contraction and αSMA expression in the wound area compared to NVM+hFF, NVM+pFF and Acell-NVM treatment groups. These findings indicate that fetal dermal cells behave differently from autologous dermal fibroblasts in excised full-thickness wounds. Several factors can account for the different behavior of these multipotent fetal dermal cells. Possible factors that play a role at an inflammatory level will be discussed first. Second, we will discuss the influence of fetal dermal cells on αSMA expression, and finally the limitations of this study.

The increased inflammatory response in wounds transplanted with either hFFs or pFFs is in contrast to the described hypo-immunogenic characteristics of (human) fetal MSCs (Chen et al. 2011; Le Blanc 2003). However, not all published results seem to support the hypo-immunogenic characteristics of allogeneic MSCs used in transplantations, as discussed by Griffin et al. (2013) and reported by Nauta et al. (2006). A possible explanation for the enhanced immune response towards xeno-or allo-transplanted fetal dermal cells in this study could be the induction of a host versus graft immune response. This is mainly coordinated by T lymphocytes, and in this study CD4^+^ and CD8^+^ lymphocytes tended to be increased in the treatment with NVM+hFF, and CD8^+^ lymphocytes also in the NVM+pFF group. This immune response could be triggered by antigens derived from the transplanted xenogeneic hFF or allogeneic pFF or MHC expression on these cells induced by the wound environment (Glenn and Whartenby 2014; Singer and Caplan 2011). It has been suggested by Ren et al. (2008, 2009) that immunosuppressive effects of MSCs are strongly dependent on their microenvironment. Glenn et al. (2014) observed that co-culture of active CD8^+^ lymphocytes with MSCs significantly induced the expression of MHC class I molecules on MSCs, and MSC cell death. Furthermore, the presence and concentration of inflammatory cytokines has been hypothesized to influence the polarization of MSCs towards a pro-inflammatory (MSC1) or anti-inflammatory phenotype (MSC2) (Glenn and Whartenby 2014; Singer and Caplan 2011; Wang et al. 2014). The differentiation of the fetal dermal cells towards an MSC1 type might also explain the enhanced inflammatory response. However, this needs to be further investigated.

In addition to enhanced inflammation in the case of fetal dermal cells, we observed a significant increase in αSMA expression in the wound area of the NVM+hFF group compared to NVM+AF at day 14. Similarly, wounds treated with pFFs showed a slightly elevated αSMA expression in the wound area in comparison with NVM+AF, but this seemed to be lower than NVM+hFF. This suggests that the enhanced or prolonged inflammatory response of fetal fibroblasts induces αSMA expression. Another possibility is that dermal fetal cells influence αSMA expression and transition to myofibroblasts by the secretion of growth factors and cytokines. MSCs express TGF-β1 (Barry et al. 2005; Glenn and Whartenby 2014; Yoon et al. 2010) and can also differentiate into myofibroblasts in response to TGF-β1 (Glenn and Whartenby 2014; Kim et al. 2014; Mishra and Banerjee 2011; Popova et al. 2010). Increased myofibroblast numbers and αSMA expression are hallmarks for fibrosis and scar formation.

Another reason for the lack of beneficial effects of fetal dermal cells can be that the survival time of the transplanted fetal dermal cells is limited. It has been reported that culture-expanded MSCs (mostly with bone marrow-derived MSCs) have a short survival time after transplantation (Eggenhofer et al. 2014; Nuschke 2014) and show little engraftment (Eggenhofer et al. 2014). A limited survival time of MSCs is possibly induced by the culture procedure (Eggenhofer et al. 2014) or by a pro-inflammatory wound bed environment (Nuschke 2014). Effects of the fetal dermis-derived MSCs can also be dependent on the timing of administration (Nuschke 2014; Ren et al. 2008; Shi et al. 2010; Wang et al. 2014). In this study, fetal dermal cells were directly transplanted after excision of the wound. Timing of administration is correlated with the wound bed milieu during wound healing. It has been hypothesized that the immunosuppressive action of MSCs is induced under highly pro-inflammatory conditions (Glenn and Whartenby 2014). This suggests that administration of MSCs at the peak of inflammation during the wound healing can be more effective.

In this study we were unable to establish beneficial effects of fetal dermal cells in NVM substitutes on wound healing and scar formation in a full-thickness porcine wound model in comparison to STSG, Acell-NVM and NVM+AF. Case reports and a clinical study have reported beneficial effects in burn wound healing by injection of bone marrow-derived MSCs (Bey et al. 2010; Lataillade et al. 2007; Rasulov et al. 2005; Rigotti et al. 2007; Sheng et al. 2009). Furthermore, improved scar quality was observed for hypertrophic burn scars injected with adipose-derived MSCs (Klinger et al. 2008). In contrast to our study, in those studies MSCs were directly injected in the area of interest and derived from a different source, mostly autologous adult MSCs. Differences in MSC origin and populations have been reviewed by Hass et al. (2011). In several animal studies, accelerated wound healing and reduced scar formation were observed when cutaneous wounds were transplanted with human MSCs (Stoff et al. 2009; Xue et al. 2013). However, these studies were mostly performed in rodent models in which wound healing is mainly mediated through contraction. The outcomes from these rodent models are therefore less predictive for the human wound healing process.

## Conclusion

Based on the results obtained in this study, we prefer the use of AF over that of pFF and hFF in full-thickness wounds to restore the dermal tissue function. In the literature, it is described that autologous as well as allogeneic MSCs seem to have great potential in the use of tissue regeneration. However, we were unable to prove an additional regenerative capacity of multipotent fetal dermal cells seeded in Novomaix and transplanted in excised full-thickness wounds. Differences between fetal dermis-derived MSCs and adult dermis-derived MSCs, and also between autologous versus allogeneic cells, should be further investigated before these can be used as a potential cell source for cell-based therapies. Especially, the influence of inflamed environments on the polarization of dermis-derived fetal MSCs towards a pro-inflammatory or anti-inflammatory phenotype needs to be further elucidated.
